# Supplementary material for: A Mixed Periodic Paralysis & Myotonia Mutant, P1158S, Imparts pH-Sensitivity in Skeletal Muscle Voltage-gated Sodium Channels
Source: Sci Rep. 2018 Apr 19;8:6304. doi: 10.1038/s41598-018-24719-y (PMC5908869; doi:10.1038/s41598-018-24719-y)
Supplement: Supplementary file 1 — Figure S1, Figure S2, Table S1, Table S2, Table S3, Table S4, Table S5, Table S6, Table S7 [file 41598_2018_24719_MOESM1_ESM.docx]

**A Mixed Periodic Paralysis & Myotonia Mutant, P1158S, Imparts pH-Sensitivity in Skeletal Muscle Voltage-gated Sodium Channels**

Mohammad-Reza Ghovanloo ^1^, Mena Abdelsayed ^1^, Colin H. Peters ^1^, Peter C. Ruben ^1^*

^1^ Department of Biomedical Physiology and Kinesiology, Simon Fraser University, Burnaby, Canada

* Corresponding Author: Dr. Peter C. Ruben, Department of Biomedical Physiology and Kinesiology, Simon Fraser University, 8888 University Drive, Burnaby, BC, Canada V5A 1S6.

e-mail: pruben@sfu.ca

phone: 778-782-9351

fax: 778-782-3424


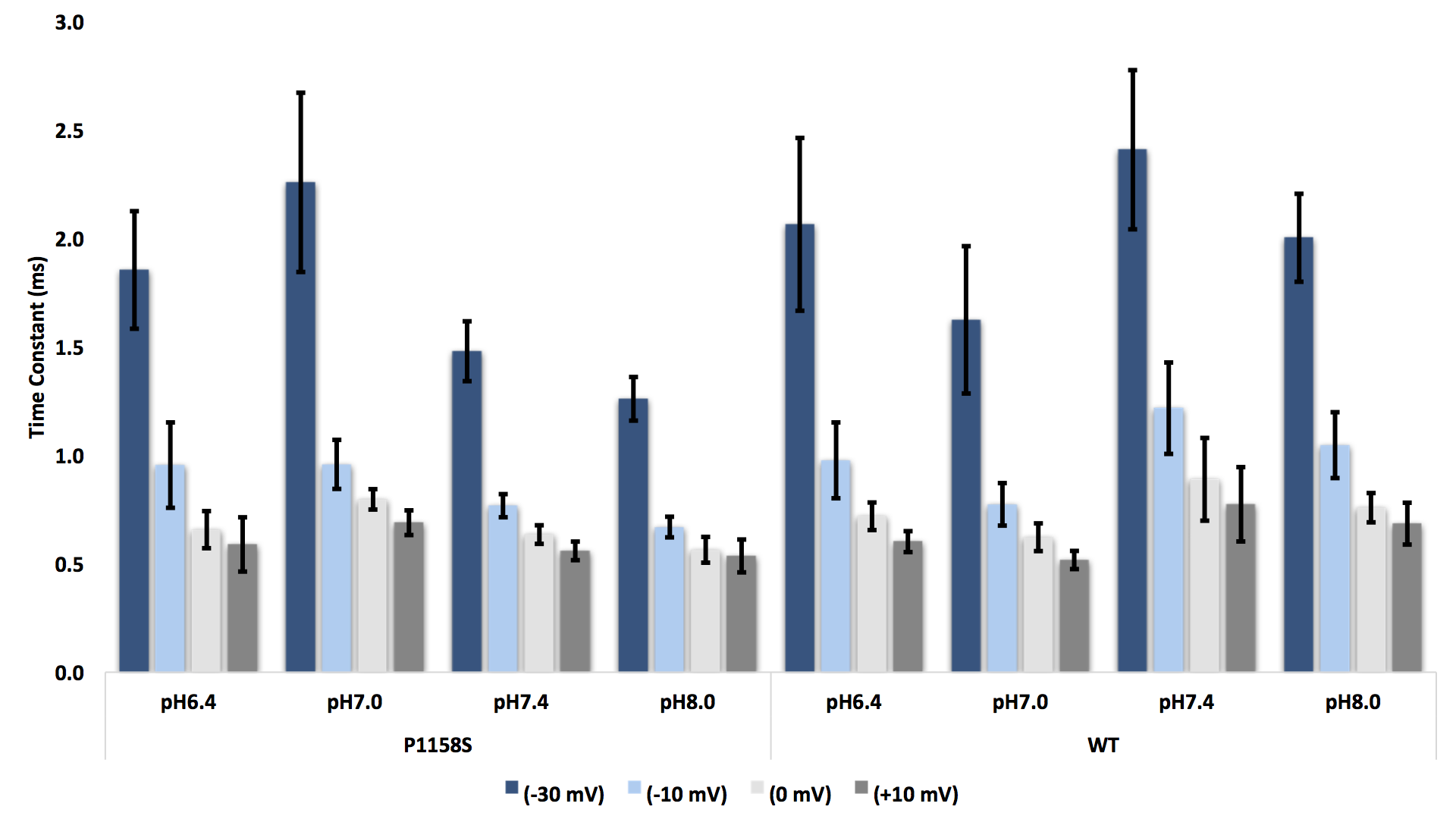


**Supplementary** **Fig. 1-** Open-state fast inactivation time constants across all conditions at: -30, -10, 0, +10 mV.


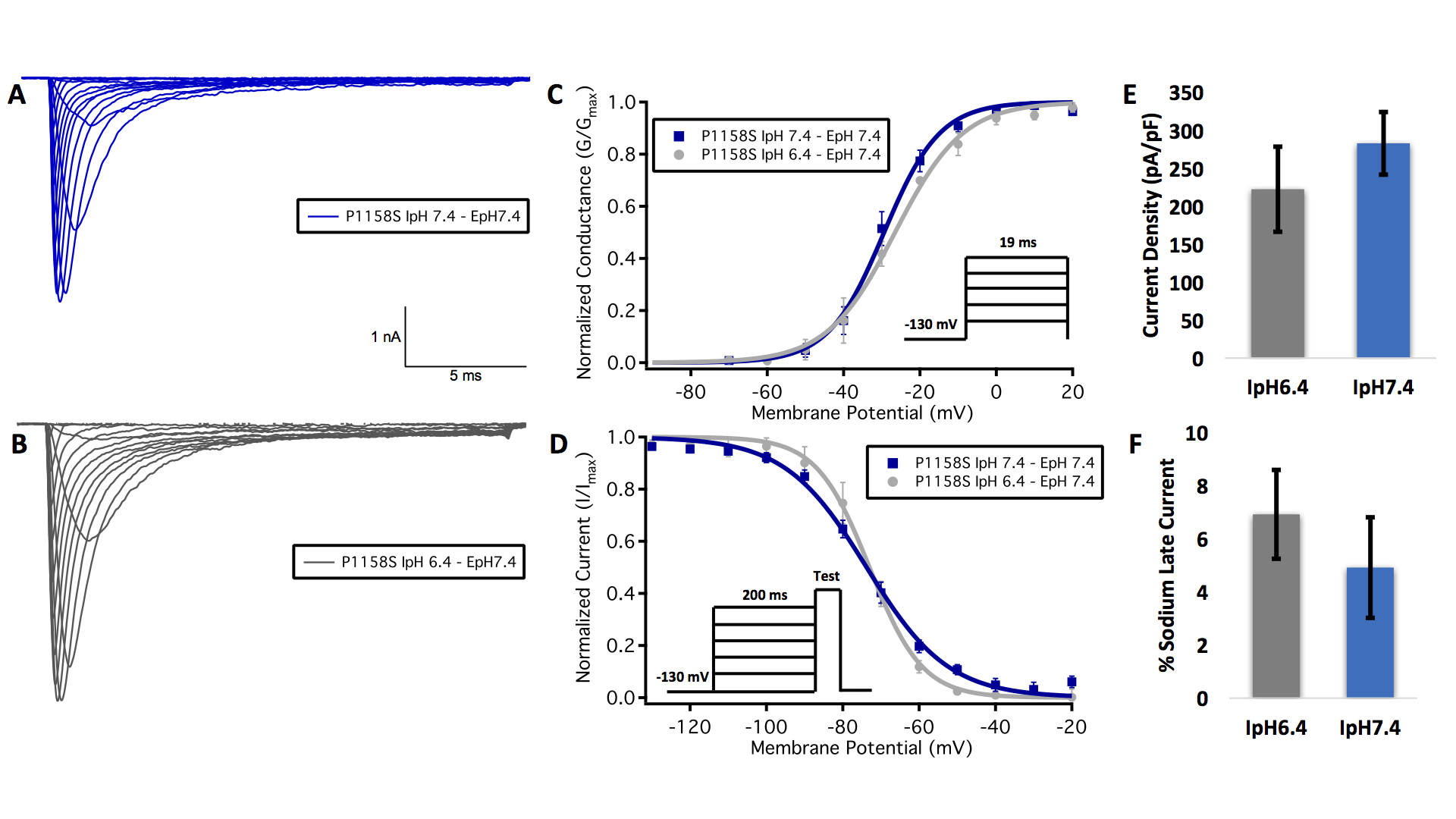


**Supplementary** **Fig. 2-** Effects of changing intracellular pH to 6.4. **(A-B)** Representative current traces of P1158S with both intra- and extracellular pH at 7.4, and intracellular pH6.4 and extracellular pH7.4, respectively. **(C)** Shows the voltage-dependence of activation for P1158S at intracellular pH7.4 (blue squares) and intracellular pH6.4 (grey circles). **(D)** Shows the voltage-dependence of steady-state fast inactivation for P1158S at intracellular pH7.4 (blue squares) and intracellular pH6.4 (grey circles). *Insets* show voltage protocols. **(E)** Shows average current density for P1158S channels at intracellular pH7.4 and 6.4. **(F)** Shows late sodium current as a percentage of peak sodium current for WT and P1158S channels.

**Supplementary** **Table 1- Conductance comparison across pH6.4 to 8.0 between WT and P1158S**

| **Channel Type-pH** | **Mean V_1/2_ ± SE (mV)** | **Mean z ± SE (slope)** | **n** |
| --- | --- | --- | --- |
| P1158S-pH6.4 | -19.48 ± 2.22^A^ | 2.76 ± 0.32 | 9 |
| P1158S-pH7.0 | -22.11 ± 2.52^A^ | 2.68 ± 0.36 | 7 |
| P1158S-pH7.4 | -29.95 ± 2.35^B^ | 4.71 ± 0.34 | 8 |
| P1158S-pH8.0 | -34.77 ± 2.35^B^ | 3.02 ± 0.34 | 8 |
| WT-pH6.4 | -25.86 ± 2.72 | 3.15 ± 0.39 | 6 |
| WT-pH7.0 | -24.87 ± 2.52 | 3.56 ± 0.36 | 7 |
| WT-pH7.4 | -24.84 ± 2.52 | 3.14 ± 0.36 | 7 |
| WT-pH8.0 | -21.92 ± 2.98 | 3.16 ± 0.43 | 5 |

Letter codes indicate statistical significance. Numbers with different letter codes are significantly different.

**Supplementary** **Table 2- Peak conductance and current densities**

| **Channel Type-pH** | **Mean density ± SE (pA/pF)** | **n** | **Mean Conductance ± SE (nS/pF)** | **n** |
| --- | --- | --- | --- | --- |
| P1158S-pH6.4 | 45.7 ± 19.5^A^ | 9 | 1.06 ± 0.45^A^ | 9 |
| P1158S-pH7.0 | 70.4 ± 20.7^A^ | 8 | 1.68 ± 0.48^A^ | 8 |
| P1158S-pH7.4 | 192.0 ± 20.7^B*^ | 8 | 3.40 ± 0.48^B*^ | 8 |
| P1158S-pH8.0 | 167.6 ± 22.1^B^ | 7 | 3.86 ± 0.51^B^ | 7 |
| WT-pH6.4 | 77.2 ± 26.1 | 5 | 1.69 ± 0.57 | 5 |
| WT-pH7.0 | 88.0 ± 26.1 | 5 | 1.90 ± 0.57 | 5 |
| WT-pH7.4 | 110.2± 18.5^*^ | 10 | 2.22 ± 0.40^*^ | 10 |
| WT-pH8.0 | 94.0 ± 23.9 | 6 | 1.78 ± 0.52 | 6 |

Letter codes and asterisk indicate statistical significance. Numbers with different letter codes and/or matching asterisks are significantly different.

**Supplementary** **Table 3- Steady-state fast inactivation comparison across pH6.4 to 8.0 between WT and P1158S**

| **Channel Type-pH** | **Mean V_1/2_ ± SE (mV)** | **Mean z ± SE (slope)** | **n** |
| --- | --- | --- | --- |
| P1158S-pH6.4 | -70.18 ± 3.16^A^ | -2.96 ± 0.25 | 6 |
| P1158S-pH7.0 | -70.95 ± 2.74^A^ | -2.53 ± 0.21 | 8 |
| P1158S-pH7.4 | -74.52 ± 2.58^B^ | -2.63 ± 0.20 | 9 |
| P1158S-pH8.0 | -84.54 ± 3.16^B^ | -2.41 ± 0.25 | 6 |
| WT-pH6.4 | -68.41 ± 3.16 | -3.74 ± 0.25 | 6 |
| WT-pH7.0 | -67.65 ± 2.74 | -3.32 ± 0.21 | 8 |
| WT-pH7.4 | -67.77 ± 2.58 | -2.49 ± 0.20 | 9 |
| WT-pH8.0 | -67.44 ± 2.74 | -3.27 ± 0.21 | 8 |

Letter codes indicate statistical significance. Numbers with different letter codes are significantly different.

**Supplementary** **Table 4- Open-state fast inactivation time constants across four voltages**

| **Channel Type-pH** | **-30 mV 𝞽 ± SE (ms)** | **-10 mV 𝞽 ± SE (ms)** | **0 mV 𝞽 ± SE (ms)** | **+10 mV 𝞽 ± SE (ms)** | **n** |
| --- | --- | --- | --- | --- | --- |
| P1158S-pH6.4 | 1.85 ± 0.27 | 0.95 ± 0.20 | 0.66 ± 0.09 | 0.59 ± 0.13 | 7 |
| P1158S-pH7.0 | 2.26 ± 0.41 | 0.96 ± 0.11 | 0.80 ± 0.05 | 0.69 ± 0.06 | 4 |
| P1158S-pH7.4 | 1.48 ± 0.14 | 0.78 ± 0.05 | 0.63 ± 0.04 | 0.56 ± 0.04 | 13 |
| P1158S-pH8.0 | 1.26 ± 0.10 | 0.67 ± 0.05 | 0.56 ± 0.06 | 0.54 ± 0.08 | 7 |
| WT-pH6.4 | 2.06 ± 0.40 | 0.98 ± 0.17 | 0.72 ± 0.06 | 0.60 ± 0.05 | 6 |
| WT-pH7.0 | 1.62 ± 0.34 | 0.77 ± 0.10 | 0.62 ± 0.06 | 0.52 ± 0.04 | 6 |
| WT-pH7.4 | 2.41 ± 0.37 | 1.22 ± 0.21 | 0.89 ± 0.20 | 0.77 ± 0.17 | 8 |
| WT-pH8.0 | 2.00 ± 0.20 | 1.05 ± 0.15 | 0.76 ± 0.07 | 0.68 ± 0.10 | 7 |

**Supplementary** **Table 5- Late sodium current percentage across pH6.4 to 8.0**

| **Channel Type-pH** | **Mean %INaL ± SE (%INaL)** | **n** |
| --- | --- | --- |
| P1158S-pH6.4 | 7.67 ± 1.42^A^ | 9 |
| P1158S-pH7.0 | 8.96 ± 1.81^A^ | 4 |
| P1158S-pH7.4 | 4.13 ± 1.21^A^ | 6 |
| P1158S-pH8.0 | 5.18 ± 1.46^A^ | 6 |
| WT-pH6.4 | 2.66 ± 1.03^B^ | 4 |
| WT-pH7.0 | 3.33 ± 1.03^B^ | 5 |
| WT-pH7.4 | 4.34 ± 0.67^B^ | 5 |
| WT-pH8.0 | 2.57 ± 0.95^B^ | 4 |

Letter codes indicate statistical significance. Numbers with different letter codes are significantly different.

**Supplementary** **Table 6- Use-dependent inactivation comparison between WT and P1158S**

| **Channel Type-pH** | **Mean 𝞽 ± SE (s)** | **n** |
| --- | --- | --- |
| P1158S-pH6.4 | 0.132 ± 0.057^A^ | 4 |
| P1158S-pH7.0 | 0.725 ± 0.238 | 8 |
| P1158S-pH7.4 | 1.643 ± 0.309 | 3 |
| P1158S-pH8.0 | 1.753 ± 0.539^B^ | 5 |
| WT-pH6.4 | 0.416 ± 0.128 | 3 |
| WT-pH7.0 | 0.849 ± 0.090 | 6 |
| WT-pH7.4 | 0.639 ± 0.290 | 4 |
| WT-pH8.0 | 0.711 ± 0.290 | 4 |

Letter codes indicate statistical significance. Numbers with different letter codes are significantly different.

**Supplementary** **Table 7- Intracellular acidosis biophysical parameters**

| **Parameter** | **P1158S IpH7.4** | **n** | **P1158S IpH6.4** | **n** |
| --- | --- | --- | --- | --- |
| Conductance V_1/2_ ± SE (mV) | -29.95 ± 2.41 | 8 | -31.67 ± 2.58 | 7 |
| Conductance z ± SE (slope) | 4.71 ± 0.56 | 8 | 3.34 ± 0.60 | 7 |
| SSFI V_1/2_ ± SE (mV) | -74.52 ± 2.43 | 9 | -77.78 ± 2.75 | 7 |
| SSFI z ± SE (slope) | -2.63 ± 0.24 | 9 | -3.10 ± 0.27 | 7 |
| Current density ± SE (pA/pF) | 282.7 ± 45.3 | 10 | 222.3 ± 50.7 | 8 |
| %INaL ± SE (%INaL) | 4.91 ± 1.72 | 6 | 6.92 ± 2.43 | 3 |
